# A digital educational resource to support and enhance effective mentorship practices of nursing students in nursing homes: a qualitative study

**DOI:** 10.1186/s12912-023-01570-9

**Published:** 2023-11-13

**Authors:** Christina T. Frøiland, Anne Marie Lunde Husebø, Ingunn Aase, Kristin Akerjordet, Kristin Laugaland

**Affiliations:** 1 SHARE – Centre for Resilience in Healthcare, Faculty of Health Sciences, University of Stavanger, Kjell Arholms Gate 41, N-4036 Stavanger, Norway; 2 Department of Public Health, Faculty of Health Sciences, University of Stavanger, Kjell Arholms Gate 41, N-4036 Stavanger, Norway

**Keywords:** Digital educational resource, Mentorship practices, Mentoring competence, Nursing home, Registered nurse mentor

## Abstract

**Background:**

There is a grooving body of evidence emphasising the need to support and enhance effective mentorship practices for nursing students in nursing home placements, including strengthening of the pedagogical competence of registered nurse mentors. Owing to the necessity for multifaceted mentoring competence and the challenges of workload registered nurses are facing, the use of flexible digital educational resources has been suggested. However, current knowledge on the effectiveness of digital educational resources in enhancing mentorship practices in nursing homes is scarce. This study aimed to explore the perception of registered nurse mentors regarding the effectiveness of a digital educational resource, particular its usability and value-in-use in supporting and enhancing mentorship practices in nursing homes.

**Methods:**

The study applied an exploratory descriptive qualitative design. Pre- and post-mentoring semi-structured focus group interviews were conducted among a total of 23 registered nurse mentors across three Norwegian nursing homes. The transcribed interviews were thematically analysed. Standards for reporting qualitative research were followed.

**Results:**

The analysis yielded one pre-mentoring theme: (1) predominant enthusiasm and satisfaction and three post-mentoring themes: (2) enhanced confidence and motivation, (3) enhanced mentoring competence in supporting the nursing students’ learning process, and (4) factors influencing the value-in-use of the digital educational resource.

**Conclusions:**

Digital educational resources support effective mentorship practices by enhancing the confidence and motivation in the mentor role and by enabling more goal-oriented supervision and assessment tailored to the learning goals of students. The implementation of digital educational resources to support and enhance effective mentorship practices is an important avenue for further research towards achieving high-quality learning environments in clinical nursing education in general and nursing homes. Based on the study findings, nursing educational institutions should consider offering digital educational resources to develop, support, and advance mentorship training, which may more effectively impact and improve the quality of clinical nursing education.

**Supplementary Information:**

The online version contains supplementary material available at 10.1186/s12912-023-01570-9.

## Background

There is a grooving body of evidence emphasising the need for educational methods to support and enhance the pedagogical and supervisory competence of registered nurse (RN) mentors (e.g. clinical nurses) to promote effective mentorship practices [1–3]. Digital educational resources (DERs) (e.g. e-learning, online learning, web-based programmes) present new possibilities for supporting mentorship practices in clinical nursing education [4]. For example, online and virtual learning platforms have increasingly been used globally as an effective learning approach for nursing professional development, presenting more flexibility, accessibility, and efficiency [5, 6]. Research on the effectiveness of DERs related to mentorship practices of nursing students in clinical education shows that DERs promote the teaching confidence, self-efficacy, and motivation of RN mentors towards mentoring of students and facilitate more tailored, individualised supervision and assessment [7, 8].

Mentorship in nursing homes somewhat differs from that in specialist health care settings (i.e. hospitals) [9], as nursing homes are associated with a challenging and marginal learning environment where a lack of RNs and a general staff turnover are considerable [9–11]. Moreover, research has emphasised that the mentorship practices of RNs in nursing homes are characterised by variability and uncertainty in pedagogical supervisory and assessment approaches [1, 12]. A recent study that explored mentorship practices in nursing homes found that reflective dialogues and clinical discussions intended to stimulate self-reflection among students were often related to technical skills and limited to the instructions and explanations of RN mentors [1]. Nursing students have also reported negative placement experiences in nursing homes owing to variabilities in supervision practices, including limited feedback, inadequate ground for assessment, and insufficient level of familiarity of RN mentors with the learning objectives of students [10]. Consequently, targeted and context-tailored efforts to support and enhance the pedagogical competence of RN mentors are advocated and emphasised to enhance the quality of and foster enriched placement in clinical education in nursing homes [1, 10, 11].

In their recent integrative literature review, Splitgerber and colleagues [13] identified strategies for creating effective clinical student learning experiences in nursing homes. They discovered that successful student learning depends on the provision of educational resources to understand and support student learning (e.g., RN mentorship programmes, supportive academic partnership) and effective supervision arrangements (e.g., positive learning environment, constructive feedback). As the future nursing force will increasingly be working with older adults (e.g. in long-term care facilities), it is of utmost importance to support RN mentors in their educational and supervisory roles in promoting positive learning experiences for nursing students during clinical placement [13], as this may motivate them to pursue a future career within aged care [14].

Despite an emerging body of research into educational interventions to support effective mentorship practices that promote enriched learning and positive placement experience in clinical education in nursing homes, more research is required [9]. Considering the characteristics of RN mentorship practices in nursing homes including variability and ambiguity in pedagogical supervisory approaches (e.g. use of reflective dialogues, provision of feedback) the literature stresses presently inadequate mentorship training [9] and a need for enhanced mentorship practices in this specific context [1]. Specifically, more knowledge is needed concerning the effectiveness of DERs as a tool for supporting and enhancing the pedagogical competence and mentorship practices of RN mentors within nursing homes [1], as this area of research is understudied [5, 15].

Our study strives to address this knowledge gap by aiming to explore the perception of RN mentors regarding the effectiveness of a DER, particular its usability and value-in-use in supporting and enhancing mentorship practices for first-year nursing students during clinical education in nursing homes. Usability has been considered during the developmental process of DERs [16]; however, emphasis has been placed on formally evaluating these resources in the actual context in which they will be used [17]. The research literature places great emphasis on usability and ease of use as key aspects in ensuring the best possible learning outcomes from educational resources [18, 19]. Thus, exploring the usability of DERs is essential, as usability may consequently influence the educational potential of DERs [20], which relates to the perceived value-in-use in this study. Value-in-use reflects the value of using DERs perceived by RN mentors [21]. Exploring value-in-use in various contexts such as mentorship in nursing homes may add to the comprehension on how effective mentorship practices for nursing students during clinical education in nursing homes can be enhanced and supported [e.g. [21, 22]]. The present study is a part of a larger research project aiming to improve the quality of clinical education in nursing homes [23].

The following research questions guided the study:

- (1) What characterises the first impressions of RN mentors concerning the usability of a DER designed to enhance their mentorship practices?
- (2) What characterises the value-in-use of a DER in mentorship practices as perceived by RN mentors?

## Methods

### Design and setting

Based on the study aim, an exploratory descriptive qualitative design was applied [24] to explore the experiences of RN mentors regarding the usability and value-in-use of a DER. The design is deemed suitable to gain understanding of the real-world experience and context of participants, particularly when studying areas that have previously received minimal attention [24]. Pre- and post-mentoring semi-structured focus group interviews [e.g. [25]] were conducted to address the research questions and facilitate discussions and group interactions [26]. Generally, focus groups are particularly suitable for obtaining a diverse range of information and understanding specific experiences and are potentially more culturally sensitive [27]. In compliance with the first research question, pre-mentoring focus group interviews were conducted to foster initial discussions on the usability of the DER among the participants. Conversely, post-mentoring focus group interviews were conducted to illustrate the experiences of the participants regarding the value-in-use of the DER related to their mentorship practices.

The DER was offered to RN mentors across three publicly funded nursing homes situated within a city-based municipality in western Norway in 2021. In this study, mentorship in the nursing homes referred to an 8-week (a total of 240 h) clinical placement period for first-year nursing students. A RN mentor from the nursing homes provided daily supervision and follow-up for one or two students [28]. Moreover, mentorship included the participation of RNs in three mandatory tripartite meetings together with nursing students and nurse educators: one initial meeting to plan placement learning and clarify role expectations and two formal assessment discussions (mid-term and final) to facilitate learning and assess the clinical competence of students [12]. Standards for reporting qualitative research [29] were followed for this study (Additional File 1: COREQ (COnsolidated criteria for REporting Qualitative research) Checklist).

### DER

The DER used by the RN mentors in this study was co-created with mentors from nursing homes and other key stakeholders (e.g. nursing students and nurse educators) to ensure a resource in better alignment with the needs and perspectives of RN mentors [30]. The co-creative developmental process has been previously detailed by [23] and [31]. The DER was designed to support and enhance the mentorship practices for first-year nursing students during clinical education in nursing homes. The DER was named ‘DigiVis’, a Norwegian acronym for supervision in nursing, and consists of three 10–15 min modules. Positioned within a pedagogical holistic approach, the DER was based on a theory in supervision methodology [32–34] and inspired by the theoretical learning perspective of Illeris [35].

**Fig. 1:**
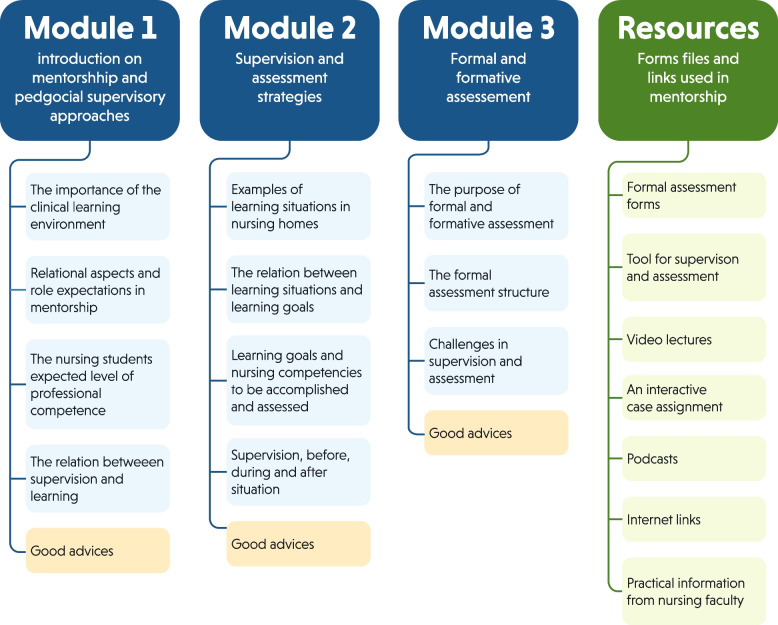
Outline of the digital educational resource content

Each module is organised with a main topic: Module 1: introduction on mentorship and pedagogical supervisory approaches (e.g. learning environment, role expectation); Module 2: supervision and assessment strategies (e.g. reflection technique, learning approach); and Module 3: formal and formative assessments. The modules consist of various components such as informative text (e.g., areas and expected levels of competence of students, learning goals, supervision, and assessment methods), illustrations, different digital supplies (e.g. ‘Tool for supervision and assessment’), podcast, video lectures, reflective activities, and an interactive case assignment. The Tool for supervision and assessment is a resource form stating the competencies nursing students are required to achieve and guides the supervisory approach of RN mentors. Based on observations of nursing students in a specific learning situation, RN mentors can assess the level of competence, professional development, and areas for further growth in conjunction with different areas of nursing competence of students. All modules end with a list of appropriate advice tailored to RN mentors, summarising key module contents. Moreover, the DER contains all practical information from the nursing faculty, which RN mentors usually receive as paper files. The DER could be employed from all kinds of digital devices (e.g. desktop computers, laptops, smartphones). In this study, tablets were also distributed to the enrolled participants prior to the pre-mentoring focus group interviews. An overview of the DER content is illustrated in Fig. 1.

### Sample

A total of 29 RN mentors across three nursing homes consented to use the DER during the clinical placement of first-year nursing students in the nursing homes. Of these RN mentors, 23 consented to participate in the semi-structured focus group interviews exploring the usability and perceived value-in-use of the DER. Twenty-three RN mentors participated in the pre- mentoring focus group interviews (interviews 1–3) conducted prior to the placement period and 18 RN mentors in the post-mentoring focus group interviews (interviews 4–6) conducted after the placement period. The reasons for drop out and non-participation of five mentors in the post-mentoring interviews were not registered. In line with the research design, the participants were recruited via purposive sampling [24], considering age, gender, background, mentoring experience, and context for generating a multiple-participant perspective [36]. Approval from the nursing home management at all three study sites was obtained initially. Informational meetings were conducted at the study sites. Approximately one third of the mentors had participated in the development of the DER. All participants provided a written consent for participation. The RN mentors included 3 men and 20 women aged from 20 to over 60 years. More than two thirds of the participants had no or minimal mentoring experience (0–5 years), whereas more than half had a diverse cultural and linguistic background and foreign nursing education. Approximately a quarter of the participants had more than 10 years of mentoring experience.

### Data collection

Data were collected in February and April 2021 during one pre- and one post-mentoring focus group interview at each of the three nursing homes enrolled. All focus group interviews were audio-recorded and had an average duration of 60 min. Owing to the COVID-19 pandemic; it was not feasible to conduct face-to-face interviews at two of the three study sites. Instead, a digital platform (Teams) was applied. The pre-mentoring interviews were conducted by the first, second, and fifth authors and the post-mentoring interviews by the first, second, third, and fifth authors, all holding a nursing and education background. Semi-structured interview guides informed by the literature and previous research guided the interviews.

**Fig. 2:**
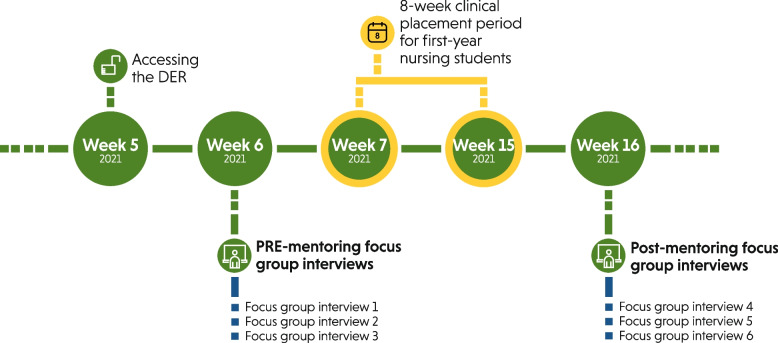
Overview of the study timeline and data collection

The participants gained access to the DER 1 week before the placement and mentorship periods (i.e. week 5). In the following week (week 6), the pre-mentoring interviews (interviews 1–3) were conducted to explore the experiences of the participants regarding the usability of the DER. After the 8-week placement period (weeks 7–15), the post-mentoring interviews (interviews 4–6) were conducted in the following week (week 16) to explore the perceived value-in-use of the DER of the participants [22]. An overview of the study timeline and data collection is illustrated in Fig. 2.

### Data analysis

The framework for thematic analyses described by Braun and Clarke [37, 38] and the research questions guided the data analysis in the following six steps: (1) familiarising with the data material, (2) generating initial codes, (3) searching for themes, (4) reviewing themes, (5) defining and naming themes, and (6) producing the report. The three pre-mentoring focus group interviews were analysed on the basis of the first research question and the three post-mentoring interviews on the basis of the second research question [39]. Table 1 illustrates examples from the data analysis. Initially, all authors independently read the complete set of transcripts to become familiar with the material and establish a sense of meaning across the six focus group interviews. Summaries of the first impressions in respect of each research question were then shared and discussed among the authors to further elaborate on distinct perceptions. In the second step, the first author [removed for peer review] generated initial codes from the data. Next, the data were interpreted, and preliminary themes were generated in respect of the research questions. In the fourth step, all authors reviewed and discussed the themes, suggesting necessary adjustments before consensus on defining and renaming the final themes was reached. In this way, the themes were created interpretably, rather than passively emerging from the coding [38]. All authors contributed to discussing and establishing the report of the findings, ensuring that the findings reflected the study aim.

**Table 1:**
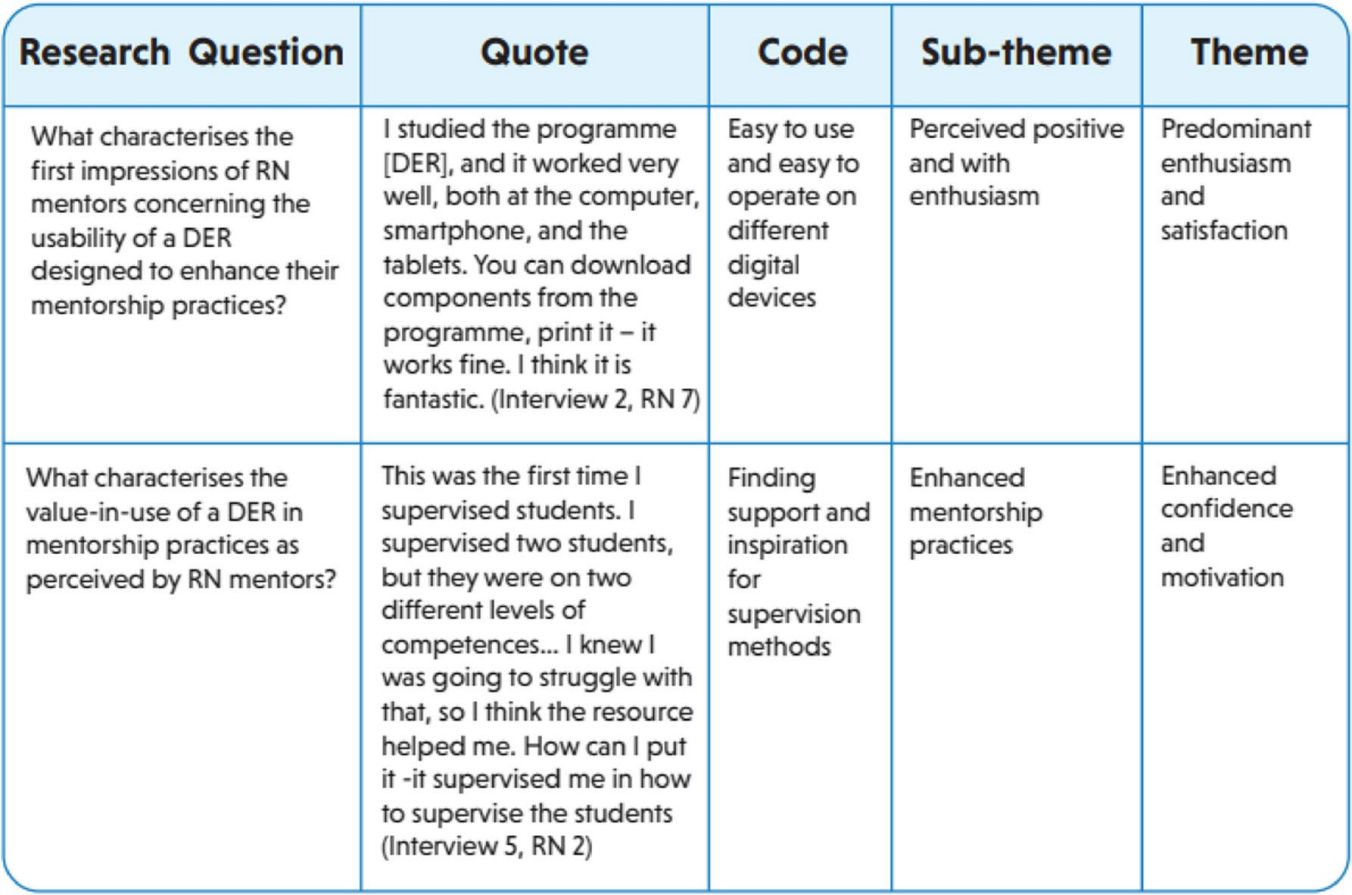
Examples from the data analysis

### Trustworthiness

The framework of quality criteria by Lincoln and Guba [40] was applied to ensure trustworthiness in the study. Credibility was ensured with the use of purposive sampling to obtain a multiple-participant perspective during data collection [41]. Contextual factors, including the type of nursing home ward, were likewise acknowledged in the analysis evaluating the use of the DER [39]. Moreover, data collection and analysis were thoroughly described in addition to the participant characteristics and the DER content [40]. The collective process of the discussions and interpretations of the findings among all authors enhanced the study dependability. More specifically, a reflective practice [38] during data analysis was featured to ensure the analytic output of the study. Further, the methodology of data collection – focus group interviews conducted pre- and post-mentoring – strengthened the quality of the study [42] by emphasising that the development in the perspective and experience of the RN mentors is vital in understanding the effectiveness of the DER in enhancing mentorship practices [e.g. [25]].

## Results

**Fig. 3:**
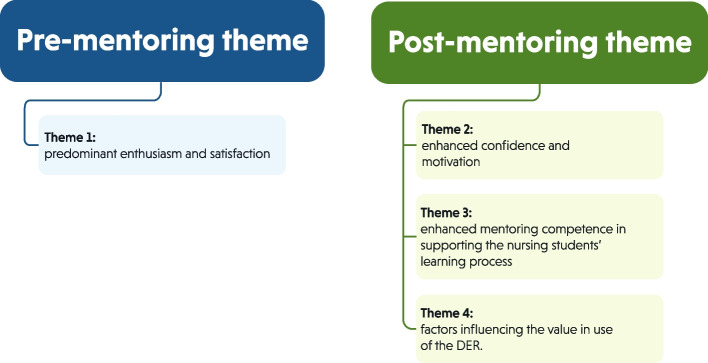
Overview of the pre-mentoring and post-mentoring themes

Derived from the analysis of the pre-mentoring interviews, one theme reflected the first impressions of the RN mentors regarding the usability of the DER: (1) predominant enthusiasm and satisfaction. The analysis of the post-mentoring interviews yielded three themes illustrating the value-in-use of the DER as perceived by the RN mentors: (2) enhanced confidence and motivation, (3) enhanced mentoring competence in supporting the nursing students’ learning process, and (4) factors influencing the value-in-use of the DER. The themes are presented in Fig. 3.

### Theme 1: predominant enthusiasm and satisfaction

The participants were predominantly enthusiastic and satisfied with the DER usability. In the pre-mentoring focus group discussions, the DER design and layout were described as appealing with the clear and appropriate structure of the modules and content components. An adequate resource functionality was highlighted by several participants, as it was easy for them to navigate searching for specific subjects. Avoiding seeking through the usual paper files from the nursing faculty was considered a great advantage. In addition to the design, layout, and ease of use, the participants related usability to the DER content and relevance for mentorship. A clear resource structure and comprehensive content added to satisfaction in pre-mentorship practices, such as being more prepared. A participant said:

*“It was nice to find answers right away. If we didn’t have the programme [the DER], we would have to wait until the nurse educator arrived to explain or go through everything.” (Interview 2, RN 2)*

The findings showed that many participants emphasised the relevance of specific components of the DER. Module 1 was described as instructive for planning the placement period, as it helped the participants to understand the mentor role and responsibility, educational context and level of competence to expect from nursing students. In Module 2, descriptions of students’ areas of competence combined with suggestions on supervision and assessment strategies were particularly engaging for many participants. The RN mentors with no or minimal mentoring experience appreciated how the DER provided them with knowledge and necessary functionalities to support the learning and development of first-year nursing students.

*“I have never supervised nursing students before. The resource [the DER] will be very helpful for me, and I so delighted to have access to it. I don’t quite know how to help the students. And it is important to know what to say to them, what to show them, and what they are supposed to learn during this placement period”. (Interview 3, RN 3)*

Satisfaction with the DER usability was particularly stressed by the inexperienced RN mentors with a diverse cultural and linguistic background. A participant explained:

*“Being a RN mentor in Norway is completely different from my home country. What I struggle with the most is assessment… It will be good for me to go through the descriptions [areas of nursing competence described in the DER] to find out what to look for when assessing a student. Reflective dialogues are also new for me. Here [in Norway], you spend a lot of time ‘reflecting on’… I am not that used to that”. (Interview 1, RN 3)*

Finally, the DER could also promote a more fair and equal treatment in the supervision and assessment of nursing students. A couple of the participants explained that dissimilarities in mentorship practices and expectations of students’ competence level might affect their learning experiences negatively.

The choice of digital devices used to access the DER differed among the participants: distributed tablets, desktop computers at the nursing home wards, private laptops at home, or mobile phones. Many participants were enthusiastic about combining devices.

*“I studied the programme [DER], and it worked very well, both at the computer, smartphone, and the tablets. You can download components from the programme, print it – it works fine. I think it is fantastic”. (Interview 2, RN 7)*

Although most RN mentors reacted with satisfaction on ease of use, a minor proportion of the participants across the age groups reported some difficulties accessing the resource as well as in downloading, saving and printing documents or specific design solutions within the DER.

### Theme 2: enhanced confidence and motivation

The second theme suggested that using the DER during the 8-week placement period enhanced the RNs’ mentoring competence. Thus, post-mentoring, the participants expressed values as gaining independence, confidence, and motivation in the mentor role from using the resource.

The participants holding no, or minimal mentoring experience emphasised various mentoring knowledge and skills gained from the DER. One of the mentors talked about a greater insight and knowledge on mentorship in general and thus gained independence when performing the mentor role. Another participant described how the DER contributed to enhancing both pedagogical competence and mentor capacity. Moreover, as a novel mentor supervising two nursing students simultaneously for the first time, one of the participants reported that the DER provided her with a better understanding of mentorship and the confidence to manage a dual mentor role.

*“This was the first time I supervised students. I supervised two students, but they were on two different levels of competences… I knew I was going to struggle with that, so I think the resource helped me. How can I put it – it supervised me in how to supervise the students”. (Interview 5, RN 2)*

Regardless of mentoring experience, several participants stressed how the DER enhanced their capability in performing core mentoring responsibilities, such as creating a supportive learning environment. Moreover, the DER enabled the participants to align personal mentorship practices from previous periods and better prepare for the mentor role.

Some participants with more than 10 years of mentoring experience related the value-in-use of the DER to recognition of the mentor role and renewed motivation in mentorship.

*“I think that it [the DER] recognises the nurse mentors’ role. And for my part, that I have become more aware of that. You get a little more professional input on what the mentor role is. It is very good for us who have supervised for a long time”. (Interview 6, RN 3)*

*“It has been more enjoyable to supervise students. Obvious because I have years of experience, but when the DER came, it got even better. I have heard about other nurses disliking mentoring students because they have not attended any mentor courses. But when this [the DER] came, I know the feedback has been very good”. (Interview 4, RN 2)*

### Theme 3: enhanced mentoring competence in supporting the nursing students’ learning process

The analysis of the post-mentoring focus group interviews indicated that the participants gained knowledge from the DER on how to support the learning process of students. This included the use of a more learning goal-orientation approach as well as reflection and assessment strategies in mentorship practices. One of the participants said:

*“I have used it [the DER] both before the students started and very actively throughout the placement period – and together with the students… The student’s professional background is important, and then it [the DER] helped me to find out what she had learnt at the nursing school and what experience she had with patients. In that way, the programme [the DER] helped me to find out where the student was [level of competence and experience], so I could tailor my supervision accordingly”. (Interview 5, RN 5)*

Several participants appreciated the DER presenting multiple ways of combining various learning situations and students’ areas of competence (e.g. ethical, pedagogical, person-centred). One of the mentors described how this was particularly useful in her ward (post-hospital care), as they usually did not have first-year nursing students. The DER enabled the mentor to identify learning situations and assign appropriate tasks corresponding to the learning goals of students.

Applying specific supervision strategies to support nursing students’ learning process was likewise discussed among the participants. One of the mentors used the DER before supervising students to gain mentorship inspiration and knowledge on how to promote reflection in supervision. Another participant used a specific situation from the nursing home ward during supervision to optimise the learning situations and encourage students in bridging theory and practice in reflective discussions.

Likewise, some of the more experienced mentors spoke about enhanced mentoring competence. However, differing from the less experienced RN mentors, these participants appreciated how the DER enabled them to enhance specific pedagogical characteristics and thus reinforce their existing mentoring competence.

*“I have done this [supervised nursing students] many times before, and I have attended nurse mentor courses. But for me it was nice to find specific thing in resource… Particularly I didn’t know a lot about assessing the students’ clinical skills, so I could look it up in the resource [the DER] for support and guidance”. (Interview 4, RN 2)*

Supporting mentor colleagues was also related to the insights gained from the DER. One of the participants talked about how mutual reflections with a colleague helped in assessing the learning and professional development of a nursing student. Another explained how DER contributed to the utilisation of available resources in the ward.

*“Me and another nurse shared a bit on the supervision since I don’t work full time. So, I used the DER on both students [my student and my colleagues’ student]. I believe that the resource can make it easier to contribute to the supervision of other nurses’ students. Because you don’t always know exactly how to handle things, but you will find it in the resource”. (Interview 4, RN 2)*

In preparing for the formal assessment discussions of students, some mentors shared that the DER improved their preparedness, involvement, and understanding of the formal assessment discussions. A participant explained how the use of the Tool for supervision and assessment provided her with a meta-perspective important to take a more active and informed position in the formal assessment discussion.

*“I used the ‘Tool for supervision and assessment’ to prepare for the formal assessment discussions. It helped me to being able to follow the assessment discussions. It helped me to understand and combine the areas of competence the student should learn and write about. I thought that was useful, I can see their professional development and what they have achieved during the period. It gave me an overview, and it was much better for me, than just observing the student and nurse educator talking, and maybe not understand what they are talking about”. (Interview 5, RN 4)*

Finally, our findings highlighted the importance of the DER content in relation to the specific nursing home context and supervision of first-year nursing students in this clinical setting. Some participants called for information on different models in supervision and challenges that can arise in mentoring nursing students. Other participants emphasised the DER content related to nursing home characteristics, such as dementia care (e.g. communication, person-centred care), important to support the learning process of students.

### Theme 4: factors influencing the value-in-use of the DER

The RN mentors reported that the lack of allocated time to supervise, insufficient level of mentoring competence, and digital illiteracy limited the perceived value-in-use of the DER. Moreover, individual student- and nurse educator-related factors (e.g., lack of interest, engagement, DER familiarity) played an influencing role.

Most participants used either all or parts of the DER but to a somewhat differing extent and mainly at the beginning of the placement period. The RN mentors with no or minimal mentoring experience used the DER on a more regular basis throughout the placement period. The participants with the longest mentoring experience reported less use of the resource. Regardless of mentoring experience, the lack of allocated time to supervise students was predominantly a negative influencing factor:

*“Honestly, I didn’t really use it [the DER] during the placement period. It was mainly before the students arrived. I viewed all of it, and I liked the practical examples [Module 2]. For 8 weeks, I have been working overtime every time I supervise students. I had to send them [the students] home to get my own chores done”. (Interview 5, RN 2)*

While some participants emphasised the DER as a useful tool in challenging mentoring situations (e.g. mentoring nursing students on a lower professional level than expected), other participants experienced the opposite. Concerns of passivity and lack of interest from students or excessive introversion and reserved behaviour of students challenged the mentors’ experience of the value-in-use. Two of the participants strived to apply the DER in mentoring culturally and linguistically diverse students. Despite being an experienced RN, one of the participants faced what she experienced as a challenging situation.

*“I had a lot of challenges with my student. It was about language and culture. So, it became too difficult to use the examples in the DER because I had to address so many other things first. I introduced the programme [DER] to the student but it was too challenging because of the language and cultural-related problems the student experienced… maybe if the placement period was longer, I could have use it [the DER]”. (Interview 4, RN 2)*

The other mentor was likewise aware of providing good and appropriate mentorship supporting the nursing student throughout the placement period. However, as the nursing student held a nursing degree from her native country, the participant strived in assessing the professional competence of the student and in meeting her current learning needs. The RN mentor did not find the necessary support in the DER.

Nurse educator-related factors influencing the value-in-use of the DER were reported by a few of the nurse mentors. Although some participants perceived the DER as helpful for holding an active position in the formal assessment discussions, others had quite different experiences. The factors limiting the value-in-use of the DER included the lack of resource familiarity of the nurse educators. Moreover, a couple of the mentors highlighted unclear role expectations between RN mentors and nurse educators – dominance of nurse educators or peripheral role of RN mentors in the assessment discussions.

## Discussion

The findings of this study suggest that DERs can act as an efficient mediating pedagogical tool to support and enhance effective mentorship practices for nursing students during clinical education in nursing homes. The findings emphasise two key aspects regarding the perceived value of the DER: enhanced mentoring competence in supporting the learning process of students and enhanced motivation and confidence in the mentor role. Another key aspect of the study findings relates to the interplay of multiple factors affecting the perceived usability of the DER related to mentoring of nursing students during clinical education within nursing homes. The discussion is structured around these key aspects of the findings along with other issues as they arise.

### Enhanced confidence, motivation, and competence in supporting students’ learning process

Consistent with previous reports [7, 8, 15], our findings suggest that a DER can support and enhance the pedagogical competence and increase the motivation and confidence of RN mentors in mentoring nursing students during clinical education in nursing homes. Other studies have highlighted similar benefits of e-learning mentoring programmes, such as improved self-efficacy and motivation towards mentoring of students [7, 8, 15]. Confidence and motivation towards being a mentor are emphasised across the literature as important drivers for effective mentoring in clinical placements [7, 8] that promote a successful mentor–student relationship and foster a supportive environment for student learning [3]. The study findings also indicate that a DER is effective in strengthening the competence of RN mentors in supporting the learning process of students and facilitating the development of professional pedagogical skills.

In this study, the DER provided the RN mentors with more efficient and accessible knowledge, overview, and description of the level of competence, educational context, background, and learning goals of students than did traditional strategies. This insight (easily accessible in the DER) was reported by the participants as highly informative and beneficial in supporting and promoting their preparedness for the supervisory role as well as in facilitating goal orientation in their mentorship practices and encouraging the use of regular reflective discussions. A goal-oriented learning path with provision of learning possibilities tailored to the learning goals of students is stressed as essential in enabling and promoting learning and professional development of students in clinical education [3, 43]. Despite this, difficulties in the interpretation of the learning goals of nursing students in clinical education have been emphasised as a persistent challenge for RN mentors across the literature, restricting their effectiveness as mentors [44]. Herein, providing the mentors with examples and advice on how to promote a reflective mentorship practice in the DER was also highlighted by the participants as instructive, making them more aware of the essential role reflection plays in supporting students’ learning process. Some participants stressed that this insight and awareness promoted the use of regular reflection in their mentorship practices, thus enhancing their pedagogical skills. Reflection as an educational strategy is considered a notable mean to reach a deeper level of exploration, enhance professional practice, and potentially improve patient outcomes [45]. Our study findings likewise suggest that a DER provides RN mentors with increased understanding of the importance of their role in formal assessment discussions, encouraging them to become more actively engaged in the assessment dialogue. Previous research has indicated that RN mentors hold a passive role in assessment discussions, which represents a barrier for the learning and development of students [1, 12].

In this study, the value of the DER was especially evident for and reported by the more inexperienced RN mentors and mentors with a diverse cultural and linguistic background. The more experienced mentors stressed the value of the DER being more related to confirmation and support of their own mentorship practices. Despite years of clinical experience, RN mentors do not necessarily achieve sufficient pedagogical teaching skills [46]. Bengtsson and Carlson [47] found that RN mentors who completed a mentorship training programme still endorsed the need for concrete tools and teaching strategies to better facilitate the learning of nursing students in clinical education. Additionally, it can be difficult to get RNs to participate in formal mentorship training programmes, as no formal competence requirements exist in Norway [10]. The literature suggests that nursing educational institutions should provide support for RN mentors in different forms [48]. DERs may more effectively support and enhance the pedagogical competence and mentorship practices of RNs, which can consequently accommodate the adverse variability and uncertainty in pedagogical supervisory approaches that characterise mentorship practices for nursing students during clinical education in nursing homes [1, 12, 49]. Taken together, our study findings suggest that a DER can act as an efficient supplementary mediating pedagogical tool to prepare RNs for their supervisory role, improve their pedagogical knowledge, enable the use of tools and resources for supervision and assessment that can support students in achieving the learning goals set for the clinical placement period, and bridge theory and practice more effectively. The increased motivation towards mentoring of students found in this study may consequently encourage RNs to attend formal mentoring programmes offered by educational institutions.

### Factors affecting the usability of a DER in clinical education in nursing homes

Overall, the DER designed to support and enhance the mentorship practices of RNs was deemed satisfactory by the participants in terms of technological and instructional design aspects, such as layout, ease of use, and relevance of content. Moreover, the flexibility and accessibility offered by the DER allowed the participants to navigate the resource easily and to read and re-read at their own pace based on their own preferences and needs [5, 50]. Both technological and instructional design aspects are vital dimensions not only in accessing and using a DER but also in motivating mentors to engage with the content in the resource [20]. Technological aspects such as ease of use and knowledge on how to use applied technology have been rated as the most significant influencing enablers for integration of digital resources as didactic tools in mentorship [18, 19]. However, our findings highlight the importance of individual and contextual factors constricting the DER usability in clinical education in nursing homes. These factors included the lack of allocated time to supervise, characteristics of RN mentors (e.g. background, mentoring experience, digital literacy), and characteristics of nursing students and nurse educators (e.g. lack of interest, learning engagement, and DER familiarity). Several participants reported using the DER mostly at the beginning of the placement period, with the lack of allocated time to supervise students as an explanatory factor reducing the DER usage. Mirroring previous reports [1, 9], the findings call for a stronger leadership commitment and acknowledgement of the influential role of RN mentors by allocating time for student supervision. Moreover, the lack of interest and engagement of students in their own learning and lack of familiarity of the nurse educators with the DER appeared to limit the motivation and thus the use of the digital resource among the RN mentors. Some participants also experienced difficulties in using the DER in particularly challenging mentoring situations. These findings indicate that usability related to instructional design also affects the motivation of students [51] and the cultural competence of mentors. Previous research [52] has emphasised a need for improving mentoring programmes related to facilitating learning in a culturally diverse workplace. Moreover, in their recent study, Oikarainen et al. [53] found that by including cultural competence in developing mentoring competence, RN mentors felt more supportive and competent in supervising culturally and linguistically diverse nursing students. Finally, the digital literacy of RN mentors has been identified as a necessary factor in ensuring successful implementation of a digital pedagogical resource [8]. In this study, some RN mentors reported difficulties in accessing the DER and in downloading and saving documents. Although earlier research has identified a link between older technology users and lower digital literacy [19], this finding was not noted in our study. Based on our findings, it is of utmost importance to develop a DER in compliance to the needs of RN mentors and the nursing home context, allocate time for supervision, and incorporate all stakeholders into the implementation of a DER to ensure transparency, engagement, and common grounds and facilitate efficiency in use. The study findings also indicate that despite a thorough co-creative developmental process involving key stakeholders [1, 31], DERs still have the potential for further development. Moreover, testing and re-testing in a real-life context are recommended to support the implementation of DERs, followed by evaluations among end-users [22].

### Limitations

Our study contributes to the evidence base on how DERs may enhance the mentorship practices of RNs in nursing homes. The strength of the study includes the inclusion of a wide variety of participants (e.g. age, experience, cultural background) mirroring a Norwegian nursing home context [36]. Furthermore, the study design allowing pre- and post-mentoring interviews emphasises the importance of highlighting usability when evaluating DERs. Despite these strengths, the study has some limitations. As the data were collected during the COVID-19 pandemic, the results may present some limitations. The data were collected partly via a digital platform (Teams). Consequently, the data quality may be limited by the inability to see visual cues and understand interactions during the focus group interviews, technical limitations with network, and digital equipment or disturbance by noise or activities where the participants were located during the interviews [54]. Likewise, one third of the mentors either completely or partly participated in developing the DER and thus were more familiar with the DER content and functionality. This may have led to a more adequate performance among these participants and thus a positive evaluation of the DER.

## Conclusions

Strengthening the quality of mentorship is paramount to promote high-quality learning environments for nursing students in clinical education. This research provides insights into the effectiveness of a DER in supporting and enhancing mentorship practices in clinical education in nursing homes. The uniqueness of the findings is relevant for clinical nursing education, as they suggest that DERs can act as an efficient mediating pedagogical tool to supplement and support effective mentorship practices for nursing students in nursing homes. However, our findings implicate several factors that challenge and constrain the usability and value-in-use of DERs in clinical education, which must be acknowledged and addressed. These factors include digital illiteracy, a lack of allocated time for supervision, a lack of student motivation and engagement, and unfamiliarity of mentors with the DER content and use. The implementation of DERs to support mentorship training is an important avenue for further research towards achieving high-quality learning environments in nursing homes. Our findings are likewise relevant for nursing educational institutions to consider integration of DERs as a supplement to face-to-face mentorship training, which may more effectively impact and improve the quality of clinical nursing education [1, 15].

### Supplementary Information

**Additional file 1.**

## Data Availability

The datasets used and analysed during the current study are available from the corresponding author on reasonable request.

## Abbreviations

RN: Registered nurse
DER: Digital educational resource
